# Comparative Analysis of Chloroplast Genome Structure and Phylogenetic Relationships Among Six Taxa Within the Genus *Catalpa* (*Bignoniaceae*)

**DOI:** 10.3389/fgene.2022.845619

**Published:** 2022-03-16

**Authors:** Feng Li, Ying Liu, Junhui Wang, Peiyao Xin, Jiangtao Zhang, Kun Zhao, Minggang Zhang, Huiling Yun, Wenjun Ma

**Affiliations:** ^1^ State Key Laboratory of Tree Genetics and Breeding, Key Laboratory of Tree Breeding and Cultivation of State Forestry Administration, Research Institute of Forestry, Chinese Academy of Forestry, National Innovation Alliance of Catalpa Bungei, Beijing, China; ^2^ Key Laboratory of National Forestry and Grassland Administration on Biodiversity Conservation in Southwest China, Southwest Forestry University, Kunming, China; ^3^ Henan Academy of Forestry, Zhengzhou, China; ^4^ Luoyang Academy of Agriculture and Forestry Sciences, Luoyang, China; ^5^ Guizhou Academy of Forestry, Guiyang, China; ^6^ Research Institute of Forestry of Xiaolongshan, Tianshui, China

**Keywords:** *catalpa*, chloroplast genome, chloroplast structure, codon bias, simple sequence repeat, phylogenetic

## Abstract

Species within the Genus *Catalpa* are mostly semievergreen or deciduous trees with opposite or whorled leaves. *C. bungei*, *C. fargesii* f. *duclouxii* and *C. fargesii* are sources of traditional precious wood in China, known as the “kings of wood”. Due to a lack of phenotypic and molecular studies and insufficient sequence information, intraspecific morphological differences, common DNA barcodes and partial sequence fragments cannot clearly reveal the phylogenetic or intraspecific relationships within *Catalpa*. Therefore, we sequenced the complete chloroplast genomes of six taxa of the genus *Catalpa* and analyzed their basic structure and evolutionary relationships. The chloroplast genome of *Catalpa* shows a typical tetrad structure with a total length ranging from 157,765 bp (*C. fargesii*) to 158,355 bp (*C. ovata*). The length of the large single-copy (LSC) region ranges from 84,599 bp (*C. fargesii*) to 85,004 bp (*C. ovata*), that of the small single-copy (SSC) region ranges from 12,662 bp (*C. fargesii*) to 12,675 bp (*C. ovata*), and that of the inverted repeat (IR) regions ranges from 30,252 bp (*C. fargesii*) to 30,338 bp (*C. ovata*). The GC content of the six chloroplast genomes were 38.1%. In total, 113 unique genes were detected, and there were 19 genes in IR regions. The 113 genes included 79 protein-coding genes, 30 tRNA genes and four rRNA genes. Five hypervariable regions (trnH-psbA, rps2-rpoC2, rpl22, ycf15-trnl-CAA and rps15) were identified by analyzing chloroplast nucleotide polymorphisms, which might be serve as potential DNA barcodes for the species. Comparative analysis showed that single nucleotide polymorphisms (SNPs) and simple sequence repeats (SSRs) were highly diverse in the six species. Codon usage patterns were highly similar among the taxa included in the present study. In addition to the stop codons, all codons showed a preference for ending in A or T. Phylogenetic analysis of the entire chloroplast genome showed that all taxa within the genus *Catalpa* formed a monophyletic group, clearly reflecting the relationships within the genus. This study provides information on the chloroplast genome sequence, structural variation, codon bias and phylogeny of *Catalpa*, which will facilitate future research efforts.

## 1 Introduction

Chloroplasts are important organelles for most higher plants and algae, allowing them to photosynthesize and convert light energy into chemical energy, and are responsible for the production of organic matter and energy storage (Nazareno et al., 2015). The chloroplast (cp) genome shows a variety of structures in cells. It is generally double-stranded and circular but may be linear, unbound to proteins, and accompanied by a complete set of replication, transcription and translation systems (Xin et al., 2020). The chloroplast genome is mainly composed of four independent structures: a large single-copy (LSC) region, a small single-copy (SSC) region, and two inverted repeat (IR) regions (IRA/B) (Liu et al., 2020; Abdullah et al., 2021a; Lee et al., 2021). The LSC and SSC regions are separated by the IR regions (Daniell et al., 2016; Gu et al., 2017). Since the chloroplast genomes of *Nicotiana tabacum* (Ohyama et al., 1986) and *Marchantia polymorpha* (Shinozaki et al., 1986) were first obtained in 1986, as of 2021, a total of approximately 4,650 sequencing records of higher plants had been added to the NCBI database. The size of the genome is generally between 120 and 160 kb, and the GC content is usually 35–40% (Cheng et al., 2017). Compared with mitochondrial and nuclear genomes, plant chloroplast genomes are more conserved in terms of structure, gene number, and gene composition, and their evolution is relatively slow, intermediate to the evolutionary rates of nuclear genomes and mitochondrial genomes (Wu et al., 2011; Dong et al., 2013). Complete chloroplast genomes are widely used for phylogenetic analysis and species identification due to their lack of recombination, small size, and high copy number per cell (Li et al., 2012; Twyford and Ness 2017; Abdullah et al., 2020a; Bi et al., 2020). Because the chloroplast genome is small and its sequence and gene composition are conserved, it is highly suitable for analyzing the systematic evolution of complex plant groups (Jansen et al., 2008; Daniell et al., 2016; Cui et al., 2019; Abdullah et al., 2020b). Studies have shown that the chloroplast genome contains additional information that can improve phylogenetic inference (Cros et al., 1998; Tong et al., 2016; Yang et al., 2018). Comparing chloroplast genome sequences provides an opportunity to discover sequence variations and identify mutation hotspots. The mutation hotspots and simple sequence repeats (SSRs) obtained from a chloroplast genome sequence can be used as effective molecular markers for identifying species and inferring population inheritance patterns (Wu et al., 2012).

*Catalpa* Scop (Bignoniaceae), an intercontinental disjunct genus, consists of ten species, with two species in eastern North America (ENA), four in eastern Asia (EAS), and four in the West Indies (WI) (Li 1952; Paclt 1952). *Catalpa* species are mostly semievergreen or deciduous trees with opposite or whorled leaves. These trees are traditional high-quality precious timber tree species in China, known as the “kings of wood”. In addition, the leaves and roots of *Catalpa* species can also be used as medicines for stomach ailments, cough, and rheumatic pain. Therefore, the development and utilization of *Catalpa* species are economically important. However, some species obtained commercially or noncommercially are mistakenly regarded as *Catalpa* species (Olsen and Kirkbride 2017). Some genes, including the inner transcribed spacer of ribosomal DNA (nrDNA ITS) and the chloroplast ndhF gene, have shown that in Bignoniaceae, *Catalpa* Scop is closely related to *Chilopsis* D. Don (Li 2008). However, due to the limited numbers of DNA fragments and variant markers in *Catalpa*, its phylogenetic relationships remain unclear (Li 2008). Further research is urgently needed to clarify the relationships between *Catalpa* species and lay a foundation for cross-breeding and drought resistance mechanism analysis. Molecular systematics has become an important method for species identification. As a source of molecular markers with more genetic information than a single gene, the chloroplast genome has been widely used in species identification (Zhao et al., 2016; Yang et al., 2020). To date, research on the chloroplast genome of *Catalpa* species has been extremely limited. In this study, we sequenced the chloroplast genomes of six taxa within the genus *Catalpa*. The purpose of this research was to 1) compare the chloroplast genomes of *Catalpa* to understand the evolution of their structure, 2) to identify a highly variable area for species identification, and 3) to clarify the phylogenetic relationships of *Catalpa*. The results provide genetic background information for hybridization breeding and drought resistance mechanism analysis of *Catalpa* species.

## 2 Materials and Methods

### 2.1 Experimental Materials and DNA Extraction

Fresh leaves of six taxa of the genus *Catalpa*, namely, *C. fargesii* f. *duclouxii* (Guiding County, Guizhou Province, China), *C. fargesii* (Tianshui City, Gansu Province, China), *C. bungei* (Luoyang, Henan Province, China), *C. ovata* (Tianshui, Gansu Province, China), *C. bungei* (Jinsiqiu) (Luoyang, Henan Province, China) and *C. fargesii* f. *duclouxii* (Huangxinzimu) (Fuquan, Guizhou Province, China), were collected. Six complete chloroplast genome sequences were deposited in GenBank with accession numbers OL628864 to OL628869 (Table 1). The samples were stored in silica gel and transported to the laboratory for low-temperature preservation (−40°C). Specimens of six taxa of the genus *Catalpa* preserved at the Institute of Forestry, Chinese Academy of Forestry, Beijing, were also examined (Table 1). Total DNA was extracted following the method of Li et al. (2013) and purified by a Wizard DNA cleanup system (Promega, Madison, WI, United States). DNA quality was assessed by spectrophotometry, and integrity was evaluated using a 1% (w/v) agarose gel (Promega, Madison, WI, United States).

**TABLE 1:** Summaries of the complete chloroplast genomes of six taxa within the genus *Catalpa*.

	*C. fargesii* f*. duclouxii*	*C. fargesii*	*C. fargesii* f*. duclouxii* (Huangxinzimu)	C. *bungei* (Jinsiqiu)	*C. bungei*	*C. ovata*
GenBank accession number	OL628868	OL628869	OL628866	OL628865	OL628867	OL628864
Raw data point no	21995192	20011296	20020162	21755074	25543980	16234746
Mapped read no	4522145	5797641	5243356	4686024	7111529	3929601
Percentage of chloroplast genome reads (%)	20.56	28.97	26.19	21.54	27.84	24.20
Chloroplast genome coverage (X)	4,289	5512	4,973	4,445	6742	3722
Plastome size (bp)	158164	157765	158164	158143	158213	158355
LSC length (bp)	84929	84599	84929	84909	84931	85004
IR length (bp)	30285	30252	30285	30285	30309	30338
SSC length (bp)	12665	12662	12665	12664	12664	12675
GC content (%)	38.10	38.10	38.10	38.10	38.10	38.10
Number of protein-coding genes	79	79	79	79	79	79
Number of tRNA genes	30	30	30	30	30	30
Number of rRNA genes	4	4	4	4	4	4

### 2.2 Sequencing, Assembly, and Annotation

Total DNA was fragmented into 350 bp fragments by ultrasound. A paired-end library was constructed by a NEBNext Ultra™ DNA library prep kit, and PE150 sequencing was performed on the Illumina HiSeq XTen platform. The NGS QC toolkit was used for quality control and to filter the low-quality reads. We used the obtained data for *de novo* assembly of the whole chloroplast genome with the GetOrganelle v1.7.5 pipeline using the following settings: F embplant_pt, -R 15, -K85 and 105. Using the published chloroplast genome of *Tecomaria capensis* (GenBank sequence acceptance number MG831880) of Bignoniaceae as a reference sequence, the Plann program (Huang and Cronk 2015) was used to annotate the chloroplast genes of *Catalpa* species. Some genes with unsuccessful or incorrect annotations were manually added in Sequin software. The structure map of the genome was first drawn using OrganellarGenomeDRAW-a (http://ogdraw.mpimp-golm.mpg.de/index.shtml) (Lohse et al., 2013) and then edited using Adobe Illustrator CS5. All chloroplast genome sequences were uploaded to the NCBI GenBank database for future reference.

### 2.3 Repeats Analyses

GMATA (Wang and Wang 2016) software was used to analyze SSRs in the chloroplast genomes of the six taxa of the genus *Catalpa* with the parameters set as 1-10, 2-4, 3-4, 4-3, 5-3 and 6-3, that is, mononucleotide SSRs with a repeat unit of 1 and a repetition number ≥10, dinucleotide SSRs with a repeat unit of 2 and a repetition number ≥6, trinucleotide SSRs with a repeat unit of 3 and a repetition number ≥5, and tetranucleotide, pentanucleotide, and hexanucleotide SSRs with a repeat unit of 4, 5, and 6, respectively, and a repetition number ≥3 (Thiel et al., 2003). Two SSR markers separated by less than 100 bp were considered a composite microsatellite. The REPuter program (Kurtz et al., 2001) was used to find forward (F), palindromic (P), reverse (R) and complementary (C) oligonucleotide repeats with a minimum repeat size of 30 bp and a similarity of 90%. The REPuter program overestimated repeats, and redundant repeats were found in large repeats as well as in duplicated tRNAs.

The six assembled chloroplast genomes were compared with MAFFT (multiple alignments using fast Fourier transform) v7 software (Katoh and Standley 2013), and then the results were manually adjusted with MEGA7 software (Kumar et al., 2016; Abdullah et al., 2021b). MEGA7 was used to quantify the mutation sites and parsimony-informative sites in the chloroplast genomes of *Catalpa*. Taking the *C. bungei* sequence as the reference, the Shuffle-LAGAN model in the mVISTA program (http://genome.lbl.gov/vista/mvista/submit.shtml) was used to analyze the whole genome of *Catalpa*. First, we manually checked for small inversions and removed them from the alignment to avoid false results. The intergenic spacer regions and protein-coding regions were extracted from the alignment in Geneious R8.1 (Kearse et al., 2012) and visualized in DnaSP v.6 to determine the nucleotide diversity of each region (Rozas et al., 2017; Abdullah et al., 2021a).

### 2.4 Codon Usage Bias Analysis

All coding sequences (CDSs) were manually extracted from the chloroplast genomes. MEGA5 was used to analyze the codon usage frequency in each of the six *Catalpa* species (Kumar et al., 2008). Relative synonymous codon usage (RSCU) reflects whether a plastid gene is in a selected state, and codons with an RSCU value >1 are defined as high-frequency codons.

### 2.5 Phylogenetic Analysis

Thirty-three chloroplast genome sequences, including six from *Catalpa* and 17 from other species of Bignoniaceae, Lentibulariaceae and Lamiaceae from GenBank, were used for phylogenetic analysis. All chloroplast genome sequences were aligned using MAFFT v7, and regions with ambiguous alignment were trimmed by Gblocks 0.91b (Castresana 2000).

Phylogenetic analysis was carried out using the maximum likelihood (ML) and Bayesian inference (BI) methods. The optimal model was identified as TVM + F + I + G4 by ModelFinder based on the Bayesian information criterion (BIC) standard (recommended by the software) (Dong Zhang et al., 2020). ML calculations were performed using IQ-tree, with sampling repeated 1,000 times. BI of the phylogenies was implemented in MrBayes (Nguyen et al., 2015). Markov chain Monte Carlo (MCMC) analysis was run for 10,000,000 generations. Trees were sampled every 1,000 generations, and the initial 25% were discarded as burn-in. Finally, the average standard deviation of the split frequencies <0.01 was verified.

## 3 Results

### 3.1 Chloroplast Genome Features

**FIGURE 1:**
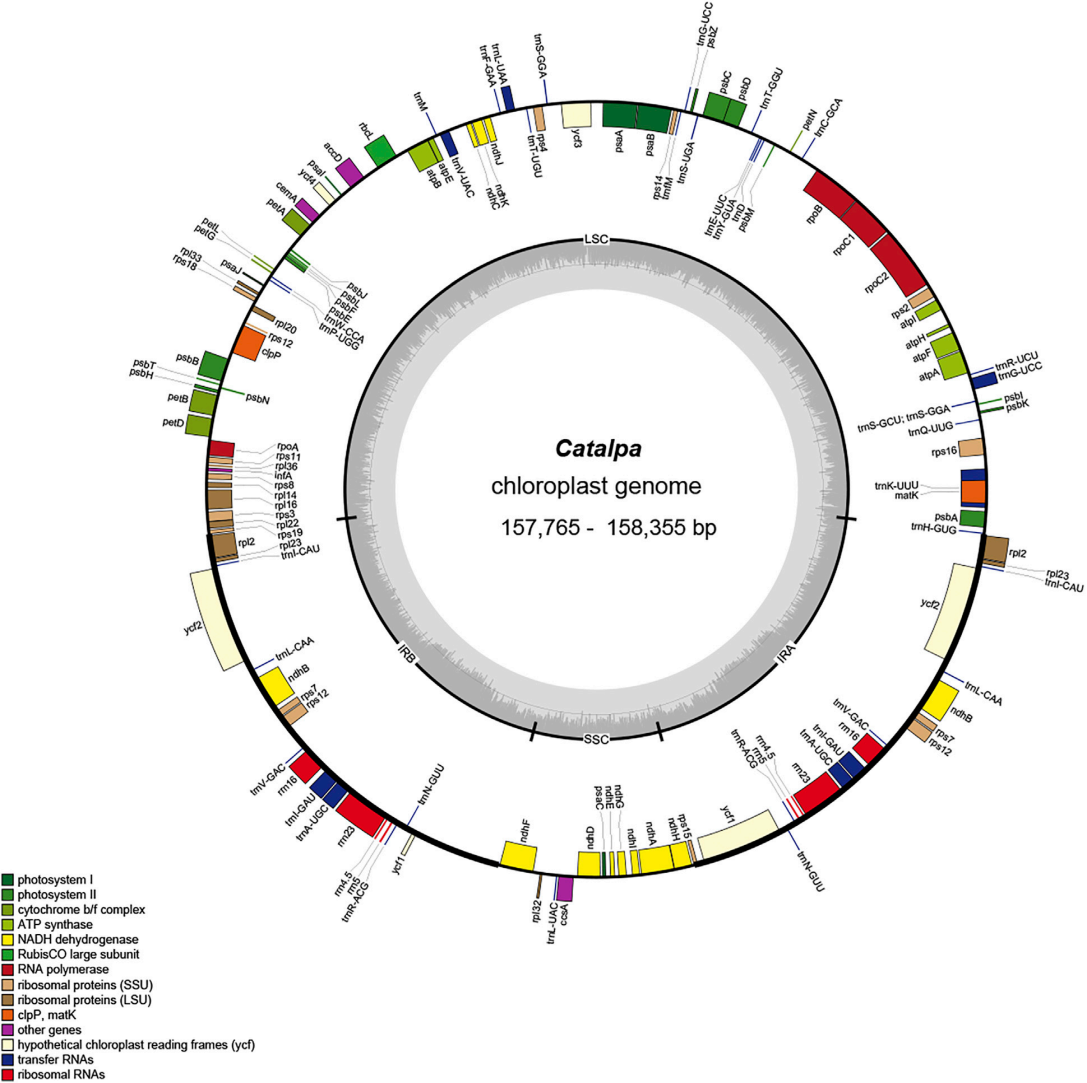
Gene map of the *Catalpa* chloroplast genome. Genes shown outside the outer circle are transcribed clockwise, and those insides are transcribed counterclockwise. Genes are color coded according to different functional groups. The darker gray in the inner circle indicates the GC content, and the lighter gray indicates the AT content. The inner circle also shows that the chloroplast genome contains two copies of inverted repeats (IRA and IRB), a large single-copy (LSC) region and a small single-copy (SSC) region. The map was constructed using OrganellarGenomeDRAW.

For the six taxa within the genus *Catalpa*, 2,435,211,900-3,831,597,000 bases of raw data with coverage ranging from 3722X-6742X were obtained (Table 1). The chloroplast genome of *Catalpa* has a typical structure, with a highly conserved, circular, double-stranded gene sequence mainly consisting of two IR regions separating two single-copy regions, namely, the LSC region and SSC region (Figure 1). The chloroplast genome length of the six taxa of the genus *Catalpa* ranged from 157,765 bp (*C. fargesii*) to 158,355 bp (*C. ovata*), the length of the LSC region ranged from 84,599 bp (*C. fargesii*) to 85,004 bp (*C. ovata*), and the length of the SSC region ranged from 12,662 bp (*C. fargesii*) to 12,675 bp (*C. ovata*) (Table 1). The length of the IR regions ranged from 30,252 bp (*C. fargesii*) to 30,338 bp (*C. ovata*). Therefore, the length variation of the LSC region was greater than that of the SSC and IR regions, and gene length variation was mainly caused by variation in the LSC region. The GC content of the genome is an important index for assessing the genetic relationships between species. The GC content of the chloroplast genomes of the six taxa within the genus *Catalpa* was 38.1%. There were almost no differences in the chloroplast genomes of the six taxa within the genus *Catalpa*.

In the chloroplast genomes of the six taxa within the genus *Catalpa*, 113 genes were detected, 19 of which were located in the IR regions. The 113 genes included 79 protein-coding genes, 30 tRNA genes and four rRNA genes (rrn5, rrn4.5, rrn16, and rrn23), and all four rRNA genes were distributed in the IR regions, resulting in a much higher GC content in the IR regions than in the two single-copy regions (Figure 1; Table 2). The infA gene was a pseudogene in all species. According to their function, the detected genes can be divided into three categories. The first category included 47 genes related to photosynthesis, including Rubisco large subunit genes, genes for components in the photosynthetic electron transport chain and genes presumed to be NAD(P)H dehydrogenase subunits. The second category consisted of six genes involved in the biosynthesis of amino acids, fatty acids and other substances, as well as some genes with unknown functions. In the third group, most of the genes were tRNA genes, including RNA polymerase subunits, rRNA and ribosomal proteins and other products. These 60 genes were related to transcription and translation. Studies have shown that introns play an important role in gene expression regulation, and many introns can enhance the level of foreign gene expression at specific times and locations in plants, in turn controlling agronomic traits (Jiao et al., 2012). Fifteen of the 113 genes contained introns, 13 contained one intron, and ycf3 and clpP contained two introns. rps12 is a spliced gene with a 5′-terminal exon located in the LSC region and a 3′-terminal exon located in the IR regions.

**TABLE 2:** Genes in the *Catalpa* chloroplast genomes.

Gene category	Gene group	Gene name
Photosynthesis-related genes	Rubisco	rbcL
	Photosystem I	psaA, psaB, psaC, psaI, psaJ
	Assembly/stability of photosystem I	**ycf3, ycf4
	Photosystem II	psbA, psbB, psbC, psbD, psbE, psbF, psbH, psbI, psbJ, psbK, psbL, psbM, psbN, psbT, psbZ
	ATP synthase	atpA, atpB, atpE, *atpF, atpH, atpI
	Cytochrome b/f complex	petA, *petB, *petD, petG, petL, petN
	Cytochrome c synthesis	ccsA
	NADPH dehydrogenase	*ndhA, *ndhB, ndhC, ndhD, ndhE, ndhF, ndhG, ndhH, ndhI, ndhJ, ndhK
Transcription- and translation-related genes	Transcription	rpoA, rpoB, *rpoC1, rpoC2
	Ribosomal proteins	rps2, rps3, rps4, rps7, rps8, rps11, rps12, rps14, rps15, rps16, rps18, rps19,*rpl2, rpl14, *rpl16, rpl20, rpl22, rpl23, rpl32, rpl33, rpl36
RNA genes	Ribosomal RNA	rrn5, rrn4.5, rrn16, rrn23
	Transfer RNA	*trnA-UGC, trnC-GCA, trnD-GUC, trnE-UUC, trnF-GAA,*trnG-UCC, *trnG-GCC, trnH-GUG, trnI-CAU, *trnI-GAU,*trnK-UUU, trnL-CAA, *trnL-UAA, trnL-UAG, trnfM-CAU, trnM-CAU, trnN-GUU, trnP-UGG, trnQ-UUG, trnR-ACG, trnR-UCU, trnS-GCU, trnS-GGA, trnS-UGA, trnT-GGU, trnT-UGU, trnV-GAC, *trnV-UAC, trnW-CCA, trnY-GUA
Other genes	RNA processing	matK
	Carbon metabolism	cemA
	Fatty acid synthesis	accD
	Proteolysis	**clpP
Genes of unknown function	Conserved reading frames	ycf1, ycf2
Pseudogenes		ycf15

Note: * represents an intron gene; ** indicates two intron genes.

### 3.2 Sequence Repeats

**FIGURE 2:**
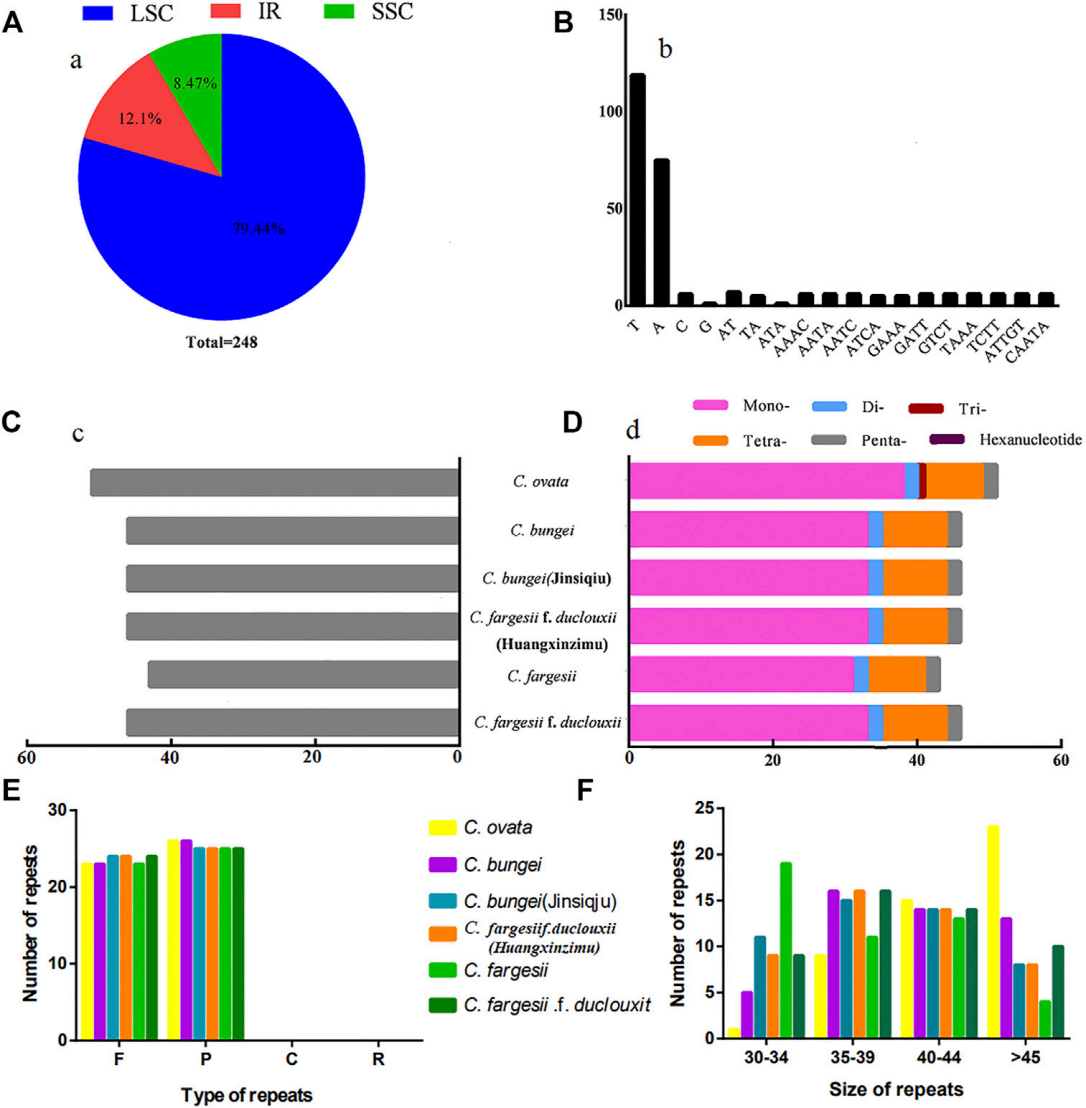
Microsatellite and oligonucleotide repeat analyses. **(A)** Frequency of identified SSRs in LSC, IR, and SSC regions. **(B)** Frequency of identified SSR motifs in different repeat class types. **(C)** Number of SSRs detected in six chloroplast genomes. **(D)** Number of SSR types detected in six chloroplast genomes. **(E)** Comparison of the various types of oligonucleotide repeats. **(F)** Comparison of repeats based on size.

SSRs, also known as microsatellites, are composed of repeating units with a length of 1-6 bp. In this study, a total of 248 SSRs were detected in the 6 chloroplast genomes of *Catalpa*. In terms of distribution, 197 SSRs were located in the LSC region (79.44%), 21 SSRs were located in the SSC region (8.47%), and 30 SSRs were located in the IR regions (12.1%). Therefore, the distribution of SSRs in the chloroplast genome of *Catalpa* is uneven (Figure 2A). The largest number of SSRs observed among the taxa within the genus *Catalpa* was 51, and the smallest was 43. The remaining four species (*C. fargesii* f. *duclouxii*, *C. fargesii* f*. duclouxii* (Huangxinzimu), *C. bungei* (Jinsiqiu), and *C. bungei*) had 46 SSR loci (Figure 2C). The chloroplast genomes of the six taxa within the genus *Catalpa* included mono-, di-, tetra-, and pentanucleotide SSRs (Figure 2D). Trinucleotide SSRs were observed in only one *Catalpa* species, and none of the six species contained hexanucleotide SSRs. Among the 248 SSR sites in the chloroplast genomes of *Catalpa* (Figure 2B), 219 sites (78.78%) were composed of A/T, and only seven sites (2.52%) contained G/C, indicating an SSR base composition preference for A/T. These findings are consistent with previous reports that SSRs are typically composed of polyadenine (PolyA) and polythymine (PolyT) repeats (Cheng et al., 2015; Shen et al., 2016). Tetranucleotides accounted for the largest percentage of SSRs (21.94%), followed by dinucleotides and pentanucleotides (both 5.04%). There were differences in the number and distribution of SSRs among the six species within the Genus *Catalpa*, which may be due to the deletion and mutation of gene sequences during the evolution of *Catalpa*. We also analyzed oligonucleotide repeats by REPuter and found four categories: palindromic (P), forward (F), reverse (R), and complementary (C). The abundance of the repeats varied among species based on the type of repeat. In the chloroplast genomes of *C. ovata*, *C. bungei*, and *C. fargesii*, REPuter revealed 49 repeats (F = 23, R = 26, P = 0, and C = 0), whereas in those of *C. bungei* (Jinsiqju), *C. fargesii* f*. duclouxi* (Huangxinzimu), and *C. fargesii* f*. duclouxi*, 49 repeats (F = 24, R = 25, P = 0, and C = 0) were detected (Figure 2E). Most of the repeats were between 35 bp to 39 bp and 40 bp to 44 bp long (Figure 2F).

### 3.3 Inverted Repeats Contraction and Expansion

**FIGURE 3:**
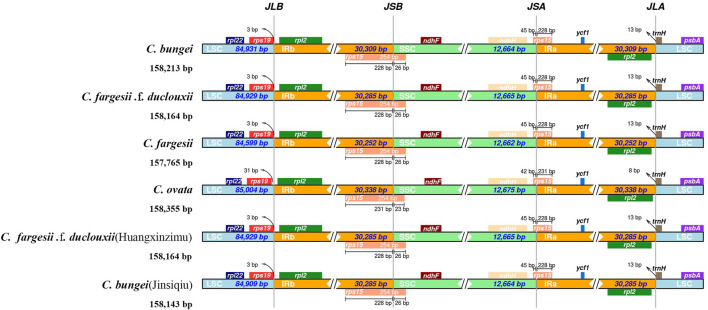
Comparisons of LSC, SSC and IR junctions among six taxa within the genus *Catalpa*.

The expansion and contraction of IR regions in the chloroplast genome are important evolutionary events in plants and relatively common phenomena, ultimately causing changes in the size and gene content of the chloroplast genome (Huang et al., 2014). To explore the potential expansion and contraction of IRs, the distributions of IR and SC border regions in the chloroplast genomes of six taxa within the genus *Catalpa* were compared. Genes with a boundary distribution of JLB, JSB, JSA and JLA included rps19, rpl2, rps15, ndHF, ndhH and trnH (Figure 3). The JLB, JSB, JSA and JLA boundaries showed very similar gene distributions. In *C. bungei*, *C. fargesii*, *C. fargesii* f*. duclouxii*, *C. fargesii* f*. duclouxii* (Huangxinzimu) and *C. bungei* (Jinsiqiu), rps19 was located in the LSC region, 3 bp from JLB, while rps19 was located 31 bp from JLB. The distributions of genes at the JLB, JSB, JSA, and JLA borders were very similar among the six taxa of the genus *Catalpa*. The length of the rps15 gene was 228 bp in the IR region of *C. bungei*, *C. fargesii*, *C. fargesii* f. *duclouxii*, *C. fargesii* Bur f. *duclouxii* (Huangxinzimu) and *C. bungei* (Jinsiqiu) but 231 bp in the IR region of *C. ovata*. Only the trnH gene of *C. ovata* was 8 bp away from JLA, and the trnH gene was located in the LSC region and 13 bp away from JLA.

### 3.4 Comparative Genomic Analysis

**FIGURE 4:**
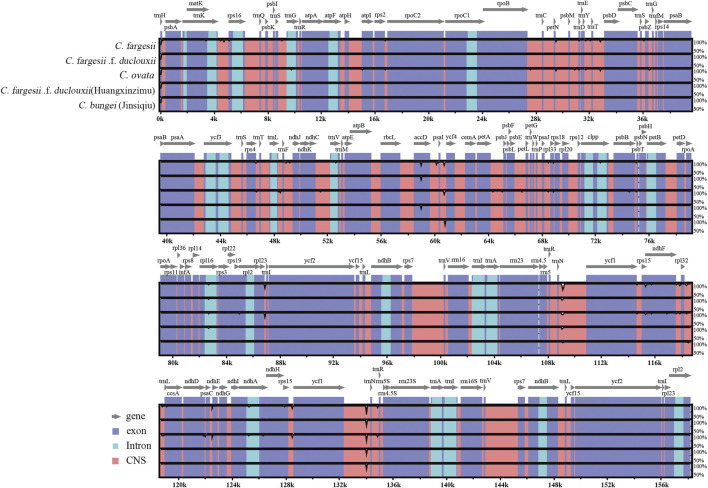
Visualization of the chloroplast genome alignments of six taxa within the genus *Catalpa* using *C. bungei* as a reference in mVISTA. The *x*-axis represents the position in the chloroplast genome. The sequence similarity of the aligned regions is shown as horizontal bars indicating the average percent identity within 50–100%.

Using the chloroplast genome of *C. bungei* as a reference, the mVISTA tool was applied to perform multiple sequence alignment, and the sequence similarity results were visualized to determine the degree of differentiation. The chloroplast genome sequences of the six taxa within the genus *Catalpa* were highly similar and conserved. The variation in the LSC and SSC regions was significantly greater than that in the IR regions, the rRNA gene was highly conserved with almost no variation, and the sequence variation in coding regions was lower than that in noncoding regions. The gene regions with large variations were accD, psaI-ycf4 and ycf1-trnN (Figure 4). The conservation degree of other genes was very high, with most of the genes being more than 90% conserved.

**FIGURE 5:**
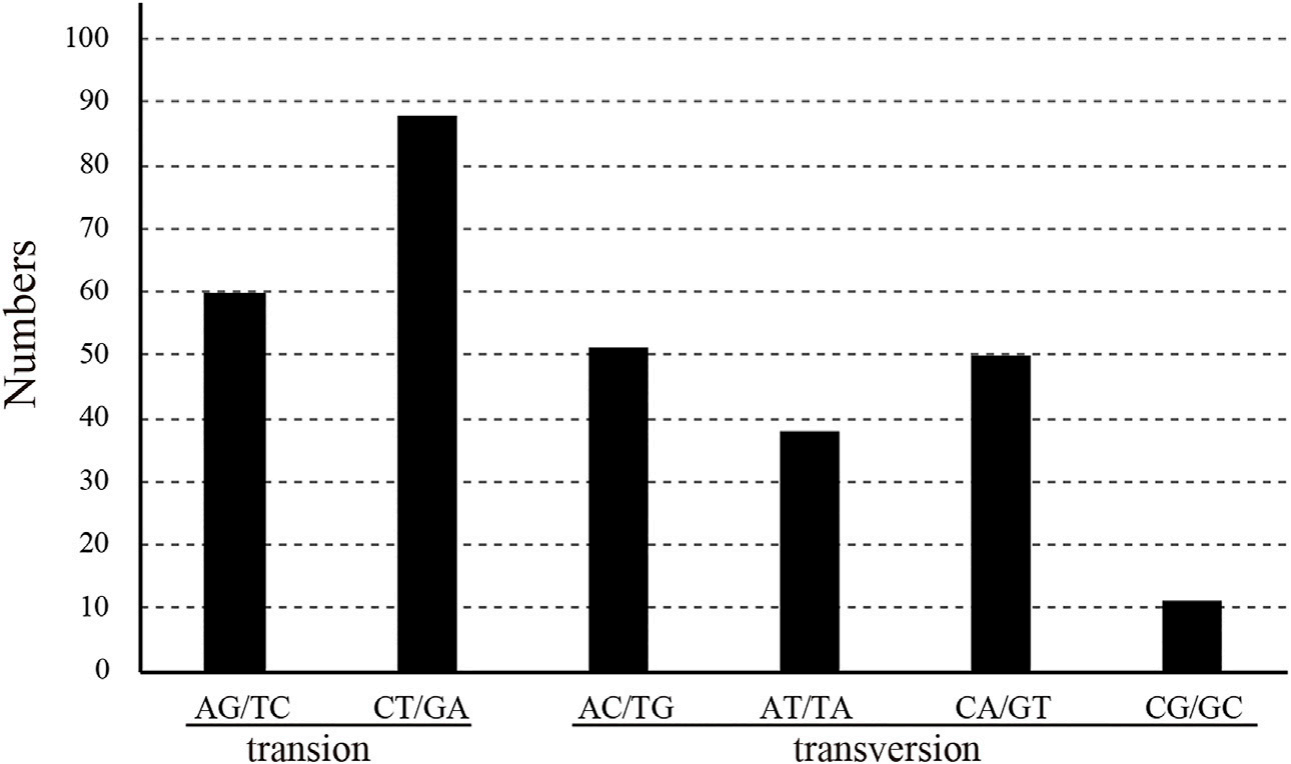
Pattern of single-nucleotide substitutions in the six *Catalpa* chloroplast genomes.

After the chloroplast genomes were compared by software, three base mutations and nondimorphic mutations were excluded from subsequent analysis. The sequence length was 159,629 bp, with a total of 301 polymorphic sites (polymorphic, S), 36 parsimony-informative sites, and six haplotypes. The nucleotide diversity (Pi) of the sequences was 0.00059 (Table 3). The IR segment had the fewest mutation sites, with 18 polymorphic sites and one parsimony-informative site. The sequence had four haplotypes, and the sequence polymorphism of this region was only 0.00018. The patterns of SNPs, 60 transitions (Ts) and 149 transversions (Tv) were determined, and the overall Ts:Tv ratio was 0.403, indicating a preference for transversions (Figure 5). The high-frequency SNPs were C to T and G to A, and mutations from A to T and from T to A exhibited the lowest frequency.

**TABLE 3:** DNA polymorphisms identified in the six *Catalpa* plastomes.

Region	Alignment length (bp)	Number of variable sites			Nucleotide polymorphism	
		Polymorphic	Singleton	Parsimony-informative	Nucleotide diversity	Haplotypes
LSC	85107	193	169	24	0.00071	6
SSC	12703	71	61	10	0.00178	5
IR	30417	18	17	1	0.00018	4
Whole plastome	159629	301	265	36	0.00059	6

**FIGURE 6:**
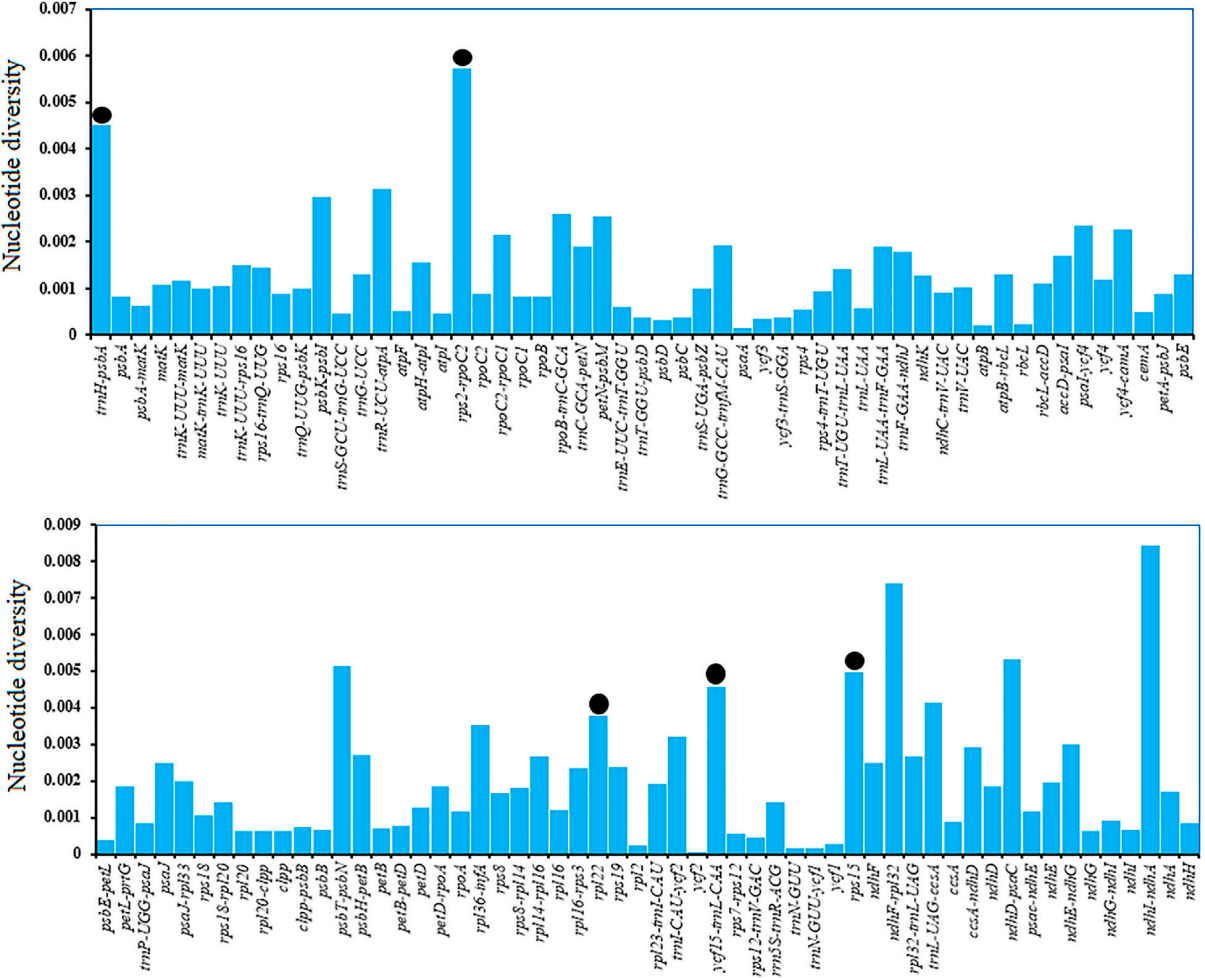
Extent of polymorphism in all plastid regions. Regions with no nucleotide diversity were excluded and are not shown here. The black circle indicates the five suitable polymorphic loci with a length >200 bp. The *x*-axis shows plastid regions, and the *y*-axis shows nucleotide diversity.

We recorded higher average polymorphism for intergenic spacer regions (0.0021) than for protein-coding sequences (0.0011). The polymorphisms of all regions are shown in Figure 6. We ignored loci <200 bp and selected 5 polymorphic regions with nucleotide diversity >0.003, of which three belonged to intergenic spacer regions and one to a protein-coding region (trnH-psbA, rps2-rpoC2, rpl22, ycf15-trnl-CAA, and rps15). rpl22 showed a nucleotide diversity of 0.00378 and contained four substitutions with 459 missing data points. A similar approach was used for ycf15-trnl-CAA, selecting a 364 bp region, which had a nucleotide diversity of 0.00458 and contained three substitutions. The selected regions may act as suitable and cost-effective markers (Table 4). These polymorphic loci might be helpful for phylogenetic inference and population genetic studies of *Catalpa* species.

**TABLE 4:** Identified suitable polymorphic loci based on comparative plastome analysis of taxa within the genus *Catalpa*.

Serial number	Region	Nucleotide diversity	Number of substitutions	Number of indels	Region length	Alignment length	Missing data (%)
1	trnH-psbA	0.00452	3	1	299	299	6.35
2	rps2-rpoC2	0.00574	3	3	214	214	2.34
3	rpl22	0.00378	4	0	459	459	0
4	ycf15-trnl-CAA	0.00458	3	0	364	364	0
5	rps15	0.00497	4	1	255	266	4.14

Abbreviation: Indels, insertions/deletions.

Note: The “Region length” data of each gene was obtained from the “*C. bungei*” reference sequence.

### 3.5 Codon Bias Analysis

**FIGURE 7:**
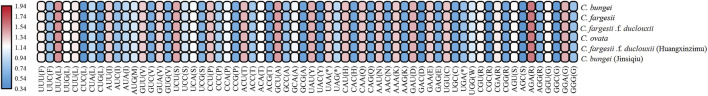
Analysis of codon bias in the chloroplast genome of *Catalpa*.

The base compositions and AT/GC contents of the six genomes of *Catalpa* were identical. Using CDSs of the chloroplast genome, we estimated the codon usage frequency of the six taxa of the genus *Catalpa*. The total number of codons detected in *C. bungei* and *C. fargesii* was 27,012 and 26,746, respectively, while the number in the other four species was 26,750 (Figure 7). The chloroplast genome of *Catalpa* encodes 20 amino acids at all codons. Leucine (Leu) was the most frequently used in the six taxa of the genus *Catalpa*, with a frequency ranging from 10.4% (2808) - 10.46% (2825), while cysteine (Cys) was the lowest in the six taxa of the genus, with a frequency reaching only 1.17% 315) - 1.18% (318). The RSCU values of the CDSs of *Catalpa* were calculated. Synonymous codon usage (RSCU value) refers to the relative probability of synonymous codons encoding corresponding amino acids for a specific codon, which eliminates the influence of amino acid composition on codon use. If there is no preference for the use of a codon, the RSCU value of the codon is equal to 1. When the RSCU value of a codon is greater than 1, it means that the codon is used more often than another, and vice versa. The results showed that the RSCU values of the six taxa included in the present study were similar. There were 30 codons with an RSCU value > 1, only one of which ended with G (UUG); the remaining 29 codons ended with A and T. The codons with an RSCU value < 1, except for UGA (stop codon) and CUA ending in A, ended in C or G. Therefore, the codon pairs ending with C and G in the *Catalpa* chloroplast genome have low bias, and they are nonpreferred codons. Due to usage frequency variation, the RSCU values of the chloroplast genome are a valuable form of evolutionary information resulting from mutation and selection that are essential in studying organismal evolution (Morton 2003).

### 3.6 Phylogenetic Analysis

**FIGURE 8:**
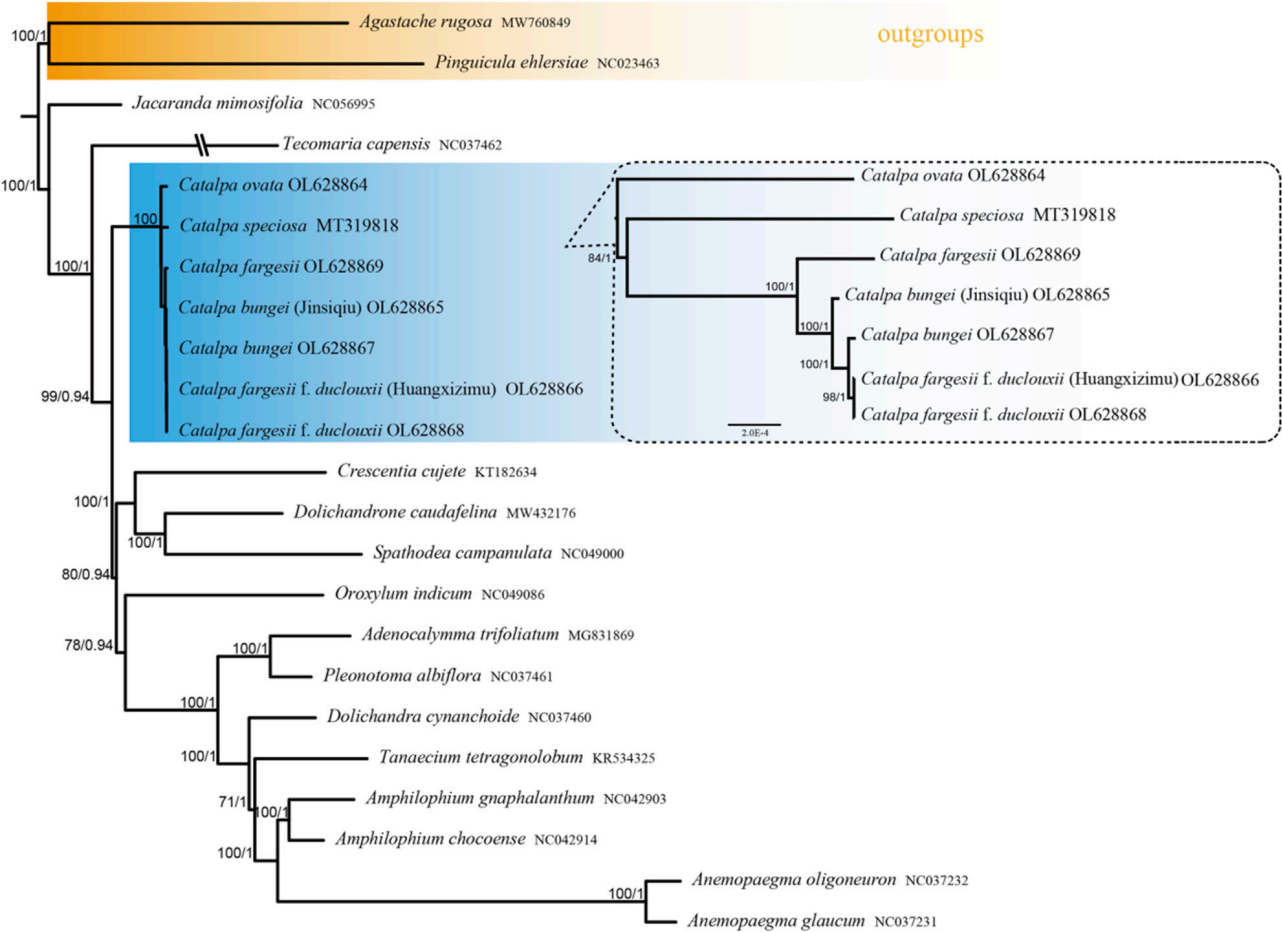
Phylogenetic tree constructed using the maximum likelihood (ML) and Bayesian inference (BI) methods based on the whole chloroplast genomes from 23 different species. The numbers above the branches represent the MI bootstrap values and Bl posterior probabilities.

The phylogenetic trees constructed using the ML and BI methods for the whole genome sequences of *Catalpa* chloroplasts had highly similar topologies (Figure 8). Strong bootstrap support and high posterior probabilities were recorded at all branch nodes. The seven species of *Catalpa* formed a monophyletic clade, with *C. ovata* diverging before the other six taxa included in the present study, with a high support rate. All taxa of the Bignoniaceae family were grouped together. In terms of the interspecific relationships of *Catalpa*, in the phylogenetic tree constructed based on the whole chloroplast genome sequences, *C. ovata* was located at the base and formed sister branches with the other six species, and *C. speciosa* formed sister branches with the other five species (*C. fargesii* f*. duclouxii*, *C. fargesii*, *C. fargesii* f*. duclouxii* (Huangxinzimu), *C. bungei* (Jinsiqiu) and *C. bungei*). *C. fargesii* f*. duclouxii* (Huangxinzimu) and C. *fargesii* Bur f. *duclouxii* formed a subbranch and were sister to C. fargesii.

## 4 Discussion

### 4.1 Chloroplast Genome of *Catalpa*

In this study, the chloroplast genomes of some taxa of the genus *Catalpa* (*C. fargesii* f*. duclouxii* (Huangxinzimu) and *C. bungei* (Jinsiqiu)) were sequenced for the first time. The chloroplast genome size ranged from 157,765 bp (*C. fargesii*) to 158,355 bp (*C. ovata*), displaying six haplotypes. There were 113 genes in the chloroplast genome of all species, including 79 protein-coding genes, 30 tRNA genes and four rRNA genes. The two genes ycf15 and ycf68 were not annotated in this study, possibly because they are pseudogenes (Lu et al., 2016; Wang et al., 2020), consistent with results reported for other *Catalpa* species (Ma et al., 2020a; Ma et al., 2020b; Li et al., 2020; Wang et al., 2020). The accD, rpl32 and ycf2 genes are lost from the chloroplast genome in some cases (Jansen et al., 2008; Oliver et al., 2010; Dong et al., 2018), but these genes were present in the *Catalpa* chloroplast genome. The overall structure of the chloroplast genome of *Catalpa* is relatively conserved, and no major gene deletions or genome rearrangements were found. The total GC content was highly consistent among species, while genome size differed slightly but not significantly. The mVISTA results and nucleotide diversity tests revealed high degrees of similarity between the chloroplast genomes, implying that the chloroplast genomes of *Catalpa* are less diverged than those of other species (Li et al., 2018).

### 4.2 Structural Variation and Codon Usage

Variation in genome structure is another form of information that helps reveal the genetic diversity of species or aspects of their population biology or evolution. The most common SSRs in the chloroplast genome of *C. ovata* were mononucleotides mainly composed of A or T and rarely G or C. Microsatellites are very important for the study of population genetics. There were significantly fewer di-, tetra-, tri-, and pentanucleotide motif repeats and no hexanucleotide repeats in the six studied species, similar to the results of Rono et al. (Rono et al., 2020). The codon is crucial to the correct expression of genetic information. In general, the start codon sequences of chloroplast genomic DNA are ATG, ATT and ATA. There were two unique patterns in RSCU and usage frequency values based on six haplotypes of protein-coding genes. First, in addition to the stop codons, all codons showed a preference for ending in A or T, but the low-frequency codons were biased toward ending in C or G. Second, the two stop codons (UAA and UGG) showed no bias, consistent with the findings of previous studies (Rono et al., 2020; Wen et al., 2021). Overall, apart from codon usage, the SNPs and SSRs of the *Catalpa* chloroplast genomes were different and can be used as excellent resources for evaluating population genetic diversity. The chloroplast genomes of the six taxa of the genus *Catalpa* showed high genetic diversity.

### 4.3 Phylogenetic Relationships

There are 10 species of *Catalpa* worldwide, with several varieties. However, due to the low genetic differentiation of *Catalpa*, the systematic relationships among these taxa are not clear (Jia et al., 2012). The pollen morphology of *C. bungei* showed that some morphological characteristics of *C. fargesii* f*. duclouxii* are the same as those of *C. fargesii* but different from those of C. bungei, which is differentiated by its morphology. The use of several chloroplast markers, such as ndhF and nuclear ribosomal DNA, for phylogenetic reconstruction is sufficient to draw firm conclusions about the interspecies relationships within *Catalpa* (Li 2008). Therefore, sampling of additional genetic features is expected to improve phylogenetic resolution. The large-scale application of Illumina HiSeq technology has improved the ability to sequence entire chloroplast genomes so that these genome sequences can be used to analyze the close relationships between species (Sheng et al., 2021; Tian et al., 2021).

In this study, we used plastome sequences to assess the phylogenetic relationships within *Catalpa*. The results revealed deep phylogenetic relationships in this genus. Dode (Dode 1907) described samples of *C. fargesii* f*. duclouxii* collected from Yunnan, China, and found that the collected samples differed from *C. fargesii* in that the undersurface of leaves and petioles were hairless. Rehder (Rehder 1913) considered *C. fargesii* f*. duclouxii* to be a variety of C. fargesii. Gilmour (Gilmour 1936) further elaborated on this view: *C. fargesii* f*. duclouxii* is a hairless variety of C. fargesii, with *C. fargesii* being more closely related than *C. bungei*. Chloroplast genome ndhF and ribosomal DNA internal transcribed spacer ITS (nrDNA ITS) sequences were used to study the interspecific relationships of *Catalpa*, and phylogenetic trees constructed with ITS and chloroplast sequences showed that *C. fargesii* f*. duclouxii* formed its own branch and formed a sister branch with *C. bungei* and *C. fargesii* (Li 2008). The results of this study show that *C. fargesii* f*. duclouxii* and *C. bungei* are more closely related, *C. fargesii* f*. duclouxii* and *C. fargesii* f. *duclouxii* (Huangxinzimu) form a branch, and the branches formed with *C. bungei* and *C. fargesii* are sister branches. The results of this study do not support the conclusion that *C. fargesii* Bur f. *duclouxii* is a variant of *C. fargesii* as proposed by Rehder (1913) and Gilmour (1936). It is suggested that *C. fargesii* f. *duclouxii* be treated as a species independent of *C. bungei* and *C. fargesii*. This study also showed that all the taxa of Bignoniaceae clustered into one group, and similar family groups formed sister branches. *Catalpa* has sufficient genetic information, and *Tecomaria capensis* (NC 037462) is closely related to *Catalpa*, consistent with the results of previous studies (Gilmour 1936; Li et al., 2020). The results of this study provide strong evidence for elucidating the evolutionary history of these species and deeply analyzing the evolutionary events of *Catalpa* and even Bignoniaceae. The further development of sequencing technology will help fully reveal the general characteristics and patterns of variation of the chloroplast genome and provide a foundation for resolving the differences between morphological and genetic classification and for obtaining an in-depth understanding of plant evolution (Xu et al., 2021). However, due to the limited number of published chloroplast genomes of taxa within the genus *Catalpa*, there are still many difficulties in phylogenetic studies of this group. In the future, more data will be needed to explore their phylogenetic relationships.

### 4.4 Oligonucleotide Repeats and Polymorphic Loci

Not all genes are phylogenetically useful in resolving taxonomic discrepancies. Oligonucleotide repeats exist widely in the plastome (Abdullah et al., 2021a). Mono-nucleotide, Palindromic, and forward repeats were the most common repeated sequences (Meng et al., 2019). Oligonucleotide repeats are also reported among the mutational events in chloroplast genomes (Abdullah, et al., 2020a). They consist of small repeats that exist in duplicate form (Kurtz et al., 2001) and mostly reported in size from 14 bp to 50 bp in chloroplast genome, unlike simple sequence repeats, which are one to six nucleotide tandem repeat units (Henriquez et al., 2014; Menezes et al., 2018; Abdullah et al., 2020a; Shahzadi et al., 2020). The results of Oligonucleotide repeats in this study are completely consistent with those mentioned above. In the taxonomy of the *Catalpa*, ndhF and the nrDNA ITS region can be discussed lower level relationships of plant groups (Baldwin et al., 1995; Soltis et al., 1998; Li 2008). However, the discriminatory power of these markers in *Catalpa* molecular phylogenetic investigations or DNA barcoding is deficient (Li 2008). Therefore, Chloroplast genome sequences provide an opportunity to elucidate patterns of genome evolution and provide valuable genetic resources for further research. Mutation events are not generally randomly distributed in the chloroplast genome but are concentrated in certain areas, forming “hotspots” (Dong et al., 2012; Wang et al., 2021). Comparing chloroplast genome sequences is an effective strategy for identifying mutation hotspots, and these highly variable regions can be used as DNA barcodes to distinguish species within specific taxa (Kuang et al., 2011; Abdullah et al., 2020a) and germplasm resources (Zhou et al., 2018; Ge et al., 2019). On the basis of the current study results, specifically, the results on nucleotide diversity among six *Catalpa* species or varieties, we suggest using a set of five divergent regions (≥200 bp) to solve taxonomic discrepancies and provide barcodes for the genus *Catalpa*. Regions of the plastome showed different polymorphisms, and certain regions were more predisposed to mutations. In this study, we identified five hypervariable regions, namely, trnH-psbA, rps2-rpoC2, rpl22, ycf15-trnl-CAA and rps15. These five regions had nucleotide diversity values of 0.00574 to 0.00378 from highest to lowest. The chloroplast genome sequences of the six taxa within the genus *Catalpa* were highly similar and conserved, and the noncoding regions had more variation than the coding regions, consistent with the results of previous studies (Perry and Wolfe 2002; Xiao-Feng Zhang et al., 2020). These variable regions can also be used to evaluate the phylogenetic relationships and interspecific differences of *Catalpa* (Yildirim et al., 2013). In this study, chloroplast genome data provided effective markers for inferring the phylogenetic relationships within *Catalpa*.

## Conclusion

In this study, the chloroplast genomes of six taxa within the genus *Catalpa* were sequenced and assembled, providing valuable genetic resources for taxa within the genus *Catalpa*. Through phylogenetic analysis of the whole chloroplast genome, the relationships within this genus were clarified for the first time. Moreover, comparative analysis of the chloroplast genomes revealed variable regions that can be used as specific DNA barcodes. The genetic resources obtained herein will contribute to studies on the population genetics, species identification, phylogenetics and conservation biology of catalpa. In the future, we will expand genome sampling, including nuclear genomes, and comprehensively assess and discuss the phylogeny and evolutionary relationships of taxa within the genus *Catalpa*.

## Data Availability

The datasets presented in this study can be found in online repositories. The names of the repository/repositories and accession number(s) can be found below: In this study has been submitted to National Center for Biotechnology Information (NCBI) (https://www.ncbi.nlm.nih.gov/) and obtained the GenBank accession number (OL628864, OL628865,OL628866, OL628867, OL628868, OL628869).
